# Association of Prescription With Body Composition and Patient Outcomes in Incident Peritoneal Dialysis Patients

**DOI:** 10.3389/fmed.2021.737165

**Published:** 2021-12-24

**Authors:** Christian Verger, Claudio Ronco, Wim Van Biesen, James Heaf, François Vrtovsnik, Manel Vera Rivera, Ilze Puide, Raymond Azar, Adelheid Gauly, Saynab Atiye, Tatiana De los Ríos

**Affiliations:** ^1^Registre de Dialyse Péritonéale de Langue Française, Pontoise, France; ^2^Department of Nephrology, Dialysis and Transplantation, International Renal Research Institute (IRRIV), San Bortolo Hospital, Vicenza, Italy; ^3^Renal Division, Ghent University Hospital, Ghent, Belgium; ^4^Zealand University Hospital, Roskilde, Denmark; ^5^Xavier Bichat Hospital, Department of Nephrology, Paris, France; ^6^Hospital Clinic de Barcelona, Barcelona, Spain; ^7^Pauls Stradins Clinical University Hospital, Riga, Latvia; ^8^Centre Hospitalier Dunkerque, Dunkerque, France; ^9^Global Medical Office, Fresenius Medical Care Deutschland GmbH, Bad Homburg, Germany

**Keywords:** lean tissue index, fat tissue index, body mass index, fluid overload, peritoneal dialysis, bioimpedance

## Abstract

**Objective:** The nutritional status of patients on peritoneal dialysis (PD) is influenced by patient- and disease-related factors and lifestyle. This analysis evaluated the association of PD prescription with body composition and patient outcomes in the prospective incident Initiative for Patient Outcomes in Dialysis–Peritoneal Dialysis (IPOD-PD) patient cohort.

**Design and Methods:** In this observational, international cohort study with longitudinal follow-up of 1,054 incident PD patients, the association of PD prescription with body composition was analyzed by using the linear mixed models, and the association of body composition with death and change to hemodialysis (HD) by means of a competing risk analysis combined with a spline analysis. Body composition was regularly assessed with the body composition monitor, a device applying bioimpedance spectroscopy.

**Results:** Age, time on PD, and the use of hypertonic and polyglucose solutions were significantly associated with a decrease in lean tissue index (LTI) and an increase in fat tissue index (FTI) over time. Competing risk analysis revealed a *U*-shaped association of body mass index (BMI) with the subdistributional hazard ratio (*HR*) for risk of death. High LTI was associated with a lower subdistributional *HR*, whereas low LTI was associated with an increased subdistributional *HR* when compared with the median LTI as a reference. High FTI was associated with a higher subdistributional *HR* when compared with the median as a reference. Subdistributional *HR* for risk of change to HD was not associated with any of the body composition parameters. The use of polyglucose or hypertonic PD solutions was predictive of an increased probability of change to HD, and the use of biocompatible solutions was predictive of a decreased probability of change to HD.

**Conclusion:** Body composition is associated with non-modifiable patient-specific and modifiable treatment-related factors. The association between lean tissue and fat tissue mass and death and change to HD in patients on PD suggests developing interventions and patient counseling to improve nutritional markers and, ultimately, patient outcomes.

**Study Registration:** The study has been registered at Clinicaltrials.gov (NCT01285726).

## Introduction

The nutritional status of patients on peritoneal dialysis (PD) is associated with patient characteristics and comorbidities, lifestyle, and treatment-associated factors (1, 2). Adequate nutritional status deserves attention as it is associated with patient outcomes (1, 3). Until now, there is no conclusive evidence, which nutritional parameter is best associated with mortality and morbidity in patients on kidney replacement therapy. Studies using body mass index (BMI) as an indicator have often found that in patients on hemodialysis (HD), the association of BMI with mortality was different than in the general population (4, 5). This apparent paradox is explained by incorrect statistical approaches mixing up association and causation, and by selection bias (6, 7).

Data on the presence of such 'reverse epidemiology' in patients on PD remain conflicting (8). In patients on PD, the nutritional status might be influenced by underlying disease state and nutritional intake, as well as chronic exposure to osmotic agents, mainly glucose. The amount of glucose being absorbed through the peritoneal membrane depends, on the one hand, on the PD prescription, such as applied glucose concentrations of the PD fluid, the fill volume, duration and number of dwells, and, on the other hand, the membrane permeability. On average, this results in an additional non-oral caloric intake ranging between 300 and 450 kcal/day (9). This carbohydrate load may have advantages and disadvantages. It can be considered as a nutritional supplement, but can also cause hyperglycemia and hyperlipidemia, and eventually lead to body weight gain (9).

Many epidemiological studies have evaluated the nutritional status and associated patient and technique survival using BMI as a nutritional marker because it is easily accessible (10, 11). Nowadays, however, it is possible to use bioimpedance spectroscopy (BIS) for a more detailed assessment of body composition (12). Besides the assessment of volume overload, BIS allows body mass to be quantified and differentiated into lean tissue and adipose tissue mass.

The Initiative for Patient Outcomes in Dialysis–Peritoneal Dialysis (IPOD-PD) study was set up as an observational study to longitudinally follow-up the fluid status together with additional patient parameters, such as nutritional status and body composition, in incident patients (13, 14). This article analyzes the evolution of nutritional status as assessed by lean tissue index (LTI) and fat tissue index (FTI) from the start of PD treatment over the first 3 years. We will associate the evolution of LTI and FTI with PD prescription patterns and this in turn with patient outcomes in terms of death and change to HD.

## Materials and Methods

### Study Objectives

The objective of this analysis is to follow-up body composition as measured by LTI and FTI in patients on incident PD for 3 years after enrollment. We further explore how these parameters are associated with PD prescribing practices, and how they may relate to the risk of death and change to HD.

### Study Design

The IPOD-PD study was an international, prospective, observational, cohort study on incident PD patients. Adult patients with chronic kidney disease who were scheduled to start PD as first kidney replacement therapy and without contraindications to routinely perform bioimpedance measurements were eligible for recruitment (14). Two years' recruitment in 135 centers in 28 countries of different geographic regions started in January 2011. Follow-up lasted until December 2015, resulting in an observation period of at least three to a maximum of 5 years, or until there was a reason for the termination of PD.

### Study Procedures and Parameters

All centers used BIS as a routine clinical practice to assess body composition. Measurements were performed with the body composition monitor (BCM; Fresenius Medical Care, Bad Homburg, Germany) (15, 16), applying multifrequency BIS through impedance measurements at 50 different frequencies from 5 kHz to 1 MHz. From these data, volume status, lean tissue mass, and fat tissue mass were calculated based on the three-compartment model described by Chamney et al. (16), which contains normohydrated lean tissue, normohydrated fat tissue, and excess fluid. Volume depletion or volume overload is calculated as the difference between the extracellular volume and the expected amount of volume in the euvolemic tissue as estimated by a previously published algorithm (15, 17), which can be expressed in absolute (L) or in relative terms (percentage of extracellular volume).

Body mass index was calculated as body weight/(body height)^2^, (kilogram/square meter), whereas LTI and FTI were calculated as lean/fat tissue mass/(body height)^2^, (kilogram/square meter).

Body composition monitor measurements performed closely before the start of PD therapy were documented, together with clinical data, laboratory parameters, planned PD prescription, clinical assessment of fluid status, and medication as baseline values. The same data were collected 1 and 3 months after the actual start of PD and then every 3 months until patients changed their renal replacement modality (transfer to HD or kidney transplantation), died, terminated the study early for other reasons or until the end of the study [see also (13)]. All data were retrieved from the patient files in the centers. The prescription of PD modality and adjustments based on BCM data collected in the study were at the discretion of the treating physician.

### Ethical Considerations

This observational study was carried out in accordance with the current version of the Declaration of Helsinki. Approval by the ethics committees and/or national authorities was received in accordance with the national regulations. Before enrollment, subjects were informed orally and in writing about the study, and written informed consent was received according to applicable law.

### Statistical Analysis

Baseline data were analyzed descriptively and are given as percentages for categorical variables, mean ± SD for normally distributed continuous variables and median (interquartile range [IQR]) for non-normally distributed continuous variables.

To analyze associations between factors measured at baseline or first month and the impact of these factors on changes in nutritional markers during the follow-up period of 3 years, a linear mixed model was applied using the SAS MIXED procedure. All available values of LTI and FTI during the 3 years were used as outcomes in the model. The variable time was calculated describing the time in months since first month and used as covariate.

For the analysis of prescription patterns, the use of hypertonic solutions was defined as applying at least one PD bag per day with a glucose concentration >1.5%. Biocompatible solutions were defined as PD fluids provided in two-chamber bags with lactate, bicarbonate, or a combination of both as buffer, and a neutral/near-neutral pH in the ready-to-use solution.

Body composition as a predictor of time to death or time to change to HD was investigated by applying a competing risk model combined with a spline analysis to consider non-linear relationships between body composition parameters and risk of death or of change to HD. Transplantation and change to HD or death were treated as competing risks. We performed Fine-Gray competing risk analysis to exclude the influence of transplantation and change to HD or death, respectively, on the cumulative incidence rate of death or change to HD (18) and computed the subdistributional hazard ratio (*HR*) with the median of BMI, LTI, and FTI as reference. This was computed with the Fine-Gray function of the survival package using R statistical software version 3.6.1 (http://cran.r-project.org).

Due to the explorative character of this observational study, no formal sample size estimation was performed; only available data were considered and no substitution procedure for missing data was applied. All analyses (except Fine-Gray competing risk analysis using splines) were performed with SAS V9.4 (SAS Institute Inc., Cary, NC, USA).

## Results

### Participants

A total of 135 centers from 28 countries recruited 1,092 participants in the study. The final analysis population consisted of *N* = 1,054 participants, as 36 patients were excluded because of breach of inclusion criteria (*n* = 2), missing follow-up visits (*n* = 6), and missing valid measurements of volume status at baseline (*n* = 30). The characteristics of the analysis population at baseline are given in Table 1.

**Table 1:** Patient characteristics.

*N*	1,054
Age [years]	58 ± 15
Gender (men) [%]	57
Height [cm]	166 ± 10
Weight [kg]	72 ± 16
**Comorbidities [%]**	
Hypertension	88
Diabetes (Type 1 + 2)	36
Cardiovascular disease (NYHA stage I, II, III, IV, unknown)	26
Liver disease	5
**Primary renal disease [%]**	
Diabetes	28
Glomerulonephritis	18
Hypertensive/large vessel disease	17
Cystic/hereditary/congenital diseases	9
Interstitial nephritis/pyelonephritis	7
Secondary glomerulonephritis/vasculitis	3
Other	7
Unknown	11

*NYHA, New York heart association*.

### PD Prescription

Peritoneal dialysis was started at a median of 30 days [IQR 19–47 days] after catheter implantation. At the start of PD, only 23% of patients were treated with automated peritoneal dialysis (APD), the proportion of which increased after 1 year to 38% and remained stable over the observation period. The proportion of patients being treated with biocompatible solutions was continually higher than 70%. In this study, 15 and 31% of patients were treated with polyglucose or at least one bag of hypertonic solution at baseline, with both proportions increasing slightly after years 1, 2, and 3. The major reasons to include polyglucose in the prescription was hydration status (51%) and dialysis dose (30%). The daily quantity of applied glucose remained somewhat stable over time in continuous ambulatory PD (CAPD), whereas in patients on APD, glucose exposure increased. Amino-acid-containing PD solutions were only prescribed to a small proportion of patients, which slightly increased over time (from 2.8% at baseline to 8.2% after 36 months); this proportion was at all time points higher in patients with diabetes than in patients without diabetes (Table 2).

**Table 2:** Peritoneal dialysis (PD) prescription.

	**Baseline**	**Year 1**	**Year 2**	**Year 3**
	***N*** **= 1,054**	***N*** **= 610**	***N*** **= 338**	***N*** **= 207**
CAPD/APD [%]	77/23	62/38	62/38	63/37
Biocomp./Bioincomp. PDF^a^ [%]	73/27	76/24	78/22	81/19
Polyglucose [%]	15	24	23	25
Hypertonic solution^b^ [%]	31	45	49	51
**Use of amino acid solution [%]**	3	5	5	8
Patients with diabetes	5	7	8	12
Patients without diabetes	2	4	3	6
Glucose applied/day, CAPD [g]	101 ± 33	106 ± 40	107 ± 38	107 ± 34
Glucose applied/day, APD [g]	135 ± 54	150 ± 62	164 ± 66	177 ± 67

a *Peritoneal dialysis fluid (PDF) prepared in two-chamber bag, with lactate, bicarbonate, or mixture of both as buffer and neutral or close to neutral pH of the ready-to-use solution*.

b *Using at least one hypertonic bag (>1.5% glucose) per day*.

*CAPD, continuous ambulatory PD; APD, automated peritoneal dialysis*.

### Nutritional Status Over Time

Nutritional status was assessed in our study through body composition monitoring and laboratory parameters (Table 3). On average, body weight and BMI increased from baseline to years 1, 2, and 3. Differentiating these changes for fat and lean tissue shows that the mean fat tissue mass and FTI increased over time, whereas mean lean tissue mass and LTI slightly decreased. Most of the changes were already present after the first year on PD, with only minor further increase or decrease at years 2 and 3.

**Table 3:** Course of nutritional parameters over time.

	**Baseline**	**Year 1**	**Year 2**	**Year 3**
		**Change from**	**Change from**	**Change from**
		**baseline^a^**	**baseline^a^**	**baseline^a^**
**Body composition parameters**				
Body weight [kg]	*N* = 1,054	*N* = 604	*N* = 337	*N* = 206
	71.92 ± 16.24	2.17 ± 5.36	2.71 ± 6.00	2.51 ± 6.23
Body mass index [kg/m^2^]	*N* = 1,054	*N* = 604	*N* = 337	*N* = 206
	25.96 ± 4.81	0.80 ± 1.97	0.99 ± 2.18	0.91 ± 2.33
Lean tissue mass [kg]	*N* = 1,046	*N* = 598	*N* = 333	*N* = 202
	37.80 ± 11.47	−0.39 ± 6.06	−0.36 ± 7.23	−1.37 ± 6.85
Lean tissue index [kg/m^2^]	*N* = 1,046	*N* = 598	*N* = 333	*N* = 202
	13.54 ± 3.28	−0.15 ± 2.19	−0.10 ± 2.59	−0.50 ± 2.48
Fat tissue mass [kg]	*N* = 1,045	*N* = 597	*N* = 333	*N* = 201
	31.84 ± 14.78	3.07 ± 7.50	3.31 ± 8.33	4.35 ± 9.12
Fat tissue index [kg/m^2^]	*N* = 1,045	*N* = 597	*N* = 333	*N* = 201
	11.61 ± 5.42	1.14 ± 2.73	1.19 ± 3.00	1.60 ± 3.39
Overhydration [L]	*N* = 1,054	*N* = 604	*N* = 337	*N* = 206
	1.87 ± 2.31	−0.58 ± 2.14	−0.35 ± 2.11	−0.34 ± 1.79
**Laboratory parameters**				
Albumin [g/L]	*N* = 961	*N* = 539	*N* = 296	*N* = 187
	37.29 ± 5.70	−1.14 ± 5.27	−1.52 ± 5.86	−1.32 ± 5.63
Creatinine [mg/dL]	*N* = 1,019	*N* = 571	*N* = 317	*N* = 197
	6.54 ± 2.54	0.93 ± 2.63	1.94 ± 3.03	2.20 ± 3.35
Hemoglobin [g/dL]	*N* = 1,013	*N* = 582	*N* = 318	*N* = 197
	10.92 ± 1.63	0.66 ± 2.03	0.40 ± 1.90	0.43 ± 2.04
CRP [mg/L]^b^	*N* = 830	*N* = 426	*N* = 241	*N* = 145
	4.14 [1.0–9.0]	0 [−2.0–2.0]	0 [−1.3–2.8]	0 [−2.0–1.1]

*Changes from baseline were calculated for those patients still in the study and with available data at respective time point*.

a *Mean change from baseline to given time point for patients still in study at given time point*.

b *Median [IQR]*.

*CRP, C-reactive protein*.

Mean serum albumin slightly decreased and mean creatinine slightly increased at follow-up visits in years 1, 2, and 3 compared with baseline. Mean hemoglobin was only marginally higher and the median of C-reactive protein (CRP) unchanged during this period (Table 3). The association of LTI with serum albumin as given in discrete categories was consistent over time. The higher levels of LTI are found in patients with higher serum albumin (Supplementary Figure 1). CRP showed the highest median value in the lowest serum albumin category at all time points (Supplementary Figure 2).

### Predictors of Change in LTI and FTI Over Time

Linear mixed models were calculated to assess the association of various patient- and treatment-related parameters with the change in LTI and FTI over time. Age, time on PD, APD vs. CAPD, and use of hypertonic solutions and polyglucose solutions were all significantly associated with a decrease in LTI over time, whereas fast, slow average, and missing data on peritoneal transport status were significantly associated with an increase in LTI over time (Table 4A). For change in FTI, nearly complementary associations were observed. Age, time on PD, use of diuretics, and use of hypertonic solutions and polyglucose solutions were all significantly associated with an increase in FTI. Among the peritoneal transport categories, all associations were not or were borderline statistically significant, except missing transport status being significantly associated with decreasing FTI (Table 4B).

**Table 4:** Linear mixed model on parameters associated with change of body composition.

**Covariate**	**Category**	**Reference**	**Estimate**	**Standard** **error**	***p***
**A: Lean tissue index [LTI, kg/m^2^]**					
Intercept			1.06	0.34	0.002
Age	Per 10 years		−0.18	0.05	<0.001
Gender	Male	Female	−0.15	0.14	0.262
Diabetes	Yes	No	0.11	0.14	0.430
Peritoneal transport status	Fast average	Slow	0.31	0.11	0.005
Peritoneal transport status	Fast	Slow	0.58	0.15	<0.001
Peritoneal transport status	Slow average	Slow	0.39	0.11	<0.001
Peritoneal transport status	Missing	Slow	0.41	0.15	0.007
Time on PD	Per month		−0.01	0.00	0.001
Diuretics (last visit)	Yes	No	−0.16	0.09	0.065
PD modality (last visit)	APD	CAPD	−0.23	0.10	0.022
Hypertonic solution (last visit)	Hypertonic agent	No hypertonic agent	−0.42	0.09	<0.001
Polyglucose (last visit)	Polyglucose	No polyglucose	−0.45	0.13	<0.001
PD solution	Bic-Buffered PDF	Lac-Buffered PDF, acidic pH	0.19	0.24	0.427
PD solution	Lac-Buffered PDF; pH neutral	Lac-Buffered PDF, acidic pH	0.14	0.18	0.439
PD solution	Bic/Lac-Buffered PDF	Lac-Buffered PDF, acidic pH	0.32	0.21	0.123
**B: Fat tissue index [FTI, kg/m^2^]**					
Intercept			−0.17	0.44	0.705
Age	Per 10 years		0.19	0.06	0.002
Gender	Male	Female	−0.11	0.17	0.513
Diabetes	Yes	No	−0.10	0.17	0.553
Peritoneal transport status	Fast average	Slow	−0.19	0.13	0.160
Peritoneal transport status	Fast	Slow	−0.36	0.18	0.050
Peritoneal transport status	Slow average	Slow	−0.15	0.13	0.265
Peritoneal transport status	Missing	Slow	−0.53	0.19	0.005
Time on PD	Per month		0.01	0.00	0.008
Diuretics (last visit)	Yes	No	0.21	0.10	0.037
PD modality (last visit)	APD	CAPD	0.21	0.12	0.094
Hypertonic solution (last visit)	Hypertonic agent	No hypertonic agent	0.49	0.10	<0.001
Polyglucose (last visit)	Polyglucose	No polyglucose	0.35	0.15	0.022
PD solution	Bic-Buffered PDF	Lac-Buffered PDF, acidic pH	−0.32	0.30	0.293
PD solution	Lac-Buffered PDF; pH neutral	Lac buffered PDF, acidic pH	−0.15	0.23	0.508
PD solution	Bic/Lac-Buffered PDF	Lac buffered PDF, acidic pH	−0.80	0.27	0.003

*A: Lean tissue index; B: Fat tissue index*.

*PD, peritoneal dialysis; CAPD, continuous ambulatory PD; APD, automated peritoneal dialysis*.

The association of the use of biocompatible PD solutions with an increase in LTI and a decrease in FTI was not statistically significant, except for lactate/bicarbonate PD solution and change in FTI (Tables 4A,B).

### Association of Body Composition With Death and Change to HD

#### Body Mass Index

**Figure 1:**
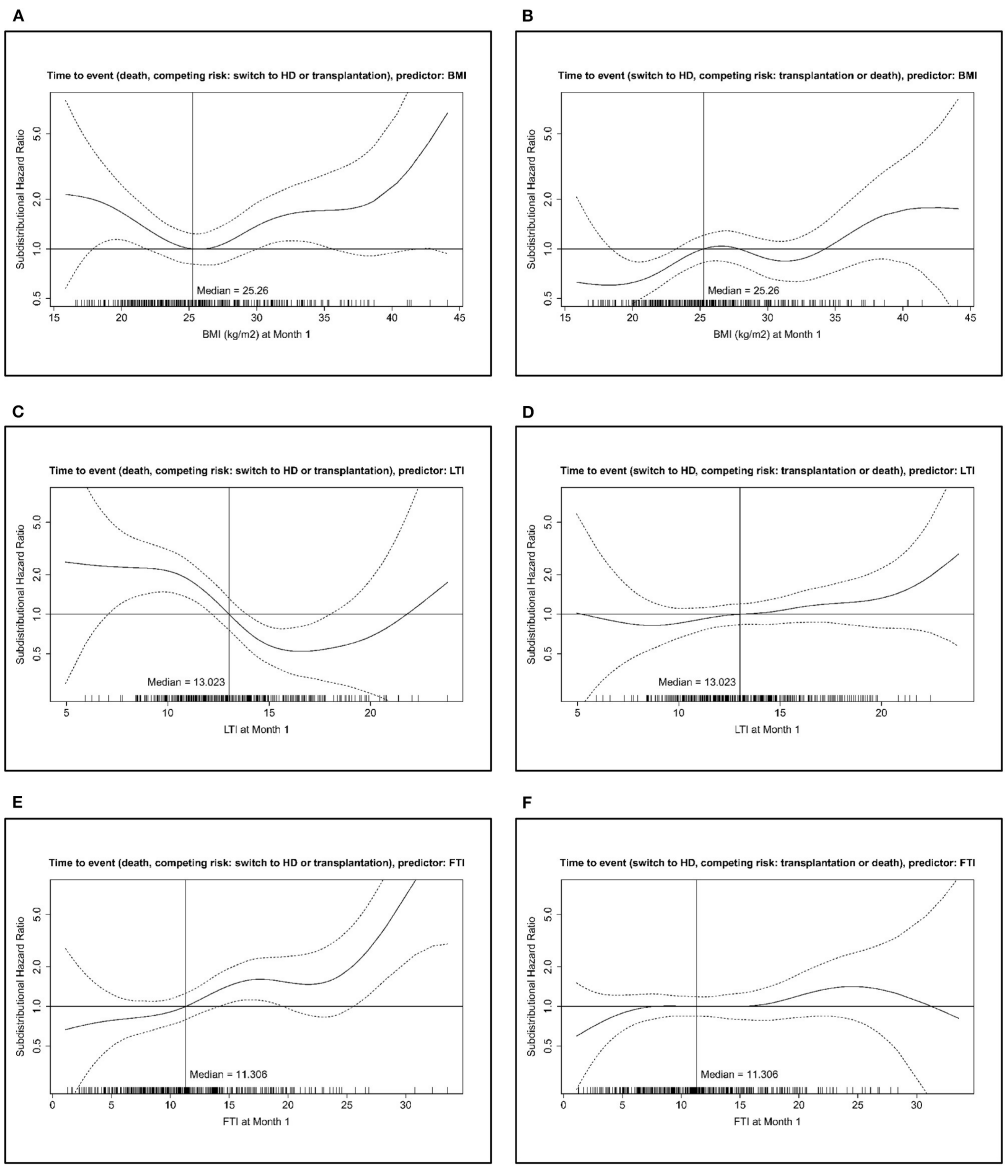
Adjusted spline analysis for the association between body composition and all-cause mortality (left) or change to hemodialysis (HD) (right). Displayed is the subdistributional hazard ratio (HR) and confidence intervals across different BMI **(A,B)**, lean tissue index (LTI) **(C,D)**, and fat tissue index (FTI) **(E,F)** levels. Adjustment was performed for age, gender, comorbidities (diabetes mellitus, cardiovascular disease, liver disease), peritoneal dialysis (PD) modality, and PD solution types.

Competing risk analysis revealed that BMI has a *U*-shaped curve of the subdistributional *HR* with increased risk of death at a BMI approximately below 22 and above 30 as compared with that of the median value of the cohort. For very low and very high BMI values, the effect was not statistically significant, probably because of the small number of patients in these ranges (Figure 1A).

The full results of the competing risk analysis (Table 5) show the impact of all covariates together with BMI on the risk of death (taking into account the competing risks change to HD and transplantation). BMI is not shown with an estimator for HR in this table as the subdistributional HR varies over the range of BMI (Figure 1). Age and presence of cardiovascular and liver diseases at baseline were associated with an increased risk of death. For overhydration and use of biocompatible solutions, there was a trend (*p* < 0.1) for an association with an increased or lower risk of death, respectively, but this did not reach statistical significance (Table 5A).

**Table 5:** Competing risk analysis on the influence of covariates together with body mass index (BMI) (A), LTI (B), FTI (C) on the event of “death” or “change to hemodialysis (HD);” BL: Baseline.

			**Death**		**Change to HD**	
**Factor**	**Category**	**Reference**	**Hazard** **ratio**	***p***	**Hazard** **ratio**	***p***
**A: Body mass index (BMI)**						
Age		Per 10 yrs	1.047	<0.001	1.003	0.609
Gender	Female	Male	1.051	0.810	0.923	0.621
Diabetes (BL)	Yes	No	1.392	0.110	0.883	0.452
Cardiovascular (BL)	Yes	No	1.956	0.001	0.968	0.855
Liver disease (BL)	Yes	No	2.141	0.036	0.844	0.662
Overhydration (L) (month 1)		Per 1 L	1.088	0.068	1.073	0.056
Modality (month 1)	CAPD	APD	0.889	0.620	0.990	0.955
Hypertonic agent (month 1)	Yes	No	1.165	0.455	1.066	0.682
Polyglucose use (month 1)	Yes	No	0.953	0.868	1.609	0.018
Biocompatible solution (month 1)	Yes	No	0.678	0.078	0.626	0.006
**B: Lean tissue index (LTI)**						
Age		Per 10 yrs	1.037	<0.001	1.006	0.276
Gender	Female	Male	0.730	0.161	1.027	0.885
Diabetes (BL)	Yes	No	1.250	0.286	0.952	0.767
Cardiovascular (BL)	Yes	No	2.014	0.001	0.990	0.953
Liver disease (BL)	Yes	No	2.267	0.024	0.805	0.575
Overhydration (L) (month 1)		Per 1 L	1.081	0.122	1.072	0.055
Modality (month 1)	CAPD	APD	0.803	0.354	0.978	0.905
Hypertonic agent (month 1)	Yes	No	1.248	0.270	1.113	0.490
Polyglucose use (month 1)	Yes	No	1.010	0.973	1.640	0.013
Biocompatible solution (month 1)	Yes	No	0.776	0.253	0.589	0.002
**C: Fat tissue index (FTI)**						
Age		Per 10 yrs	1.044	<0.001	1.003	0.600
Gender	Female	Male	0.974	0.902	0.860	0.362
Diabetes (BL)	Yes	No	1.341	0.159	0.880	0.449
Cardiovascular (BL)	Yes	No	1.876	0.002	0.954	0.795
Liver disease (BL)	Yes	No	2.402	0.015	0.809	0.585
Overhydration (L) (month 1)		Per 1 L	1.115	0.022	1.079	0.045
Modality (month 1)	CAPD	APD	0.854	0.503	0.987	0.941
Hypertonic agent (month 1)	Yes	No	1.050	0.811	1.085	0.599
Polyglucose use (month 1)	Yes	No	0.905	0.731	1.620	0.016
Biocompatible solution (month 1)	Yes	No	0.745	0.179	0.610	0.004

*BL, baseline; CAPD, continuous ambulatory PD; APD, automated peritoneal dialysis*.

For change to HD, the association with BMI was nearly complementary to that of risk of death. However, the reduced subdistributional HR was significant only at low BMI (Figure 1B).

Of the factors included in the model, the use of polyglucose was associated with an increased subdistributional HR for change to HD and use of biocompatible PD fluids with a decreased subdistributional HR for change to HD (Table 5A).

#### Lean Tissue Index

Analogous models were calculated for LTI and FTI. An LTI higher than the median value of the cohort was associated with a lower subdistributional HR of death, and an LTI below the median was associated with a higher subdistributional HR of death, as compared with the median as reference (Figure 1C).

Age and presence of cardiovascular and liver disease at baseline were associated with an increased risk of death (Table 5B). There was no clear association of LTI with the risk of change to HD (Figure 1D).

Of the factors included in the model, the use of polyglucose was associated with an increased subdistributional HR for change to HD, and use of biocompatible PD fluids was associated with a decreased subdistributional *HR* for change to HD. For overhydration, there was a trend (*p* < 0.1) for an association with an increased risk of death, but this did not reach statistical significance (Table 5B).

#### Fat Tissue Index

An FTI higher than the median value of the cohort was associated, within a certain range, with a significantly higher subdistributional HR as compared with the median as reference. The subdistributional HR for FTI below the median was not significantly different statistically to that of the median (Figure 1E).

Age, presence of cardiovascular and liver diseases at baseline, and overhydration at month 1 were associated with an increased risk of death (Table 5C).

There was no clear association of FTI with the risk of change to HD (Figure 1F).

Of the factors included in the model, overhydration and the use of polyglucose were associated with an increased subdistributional HR for change to HD and use of biocompatible PD fluids with a decreased subdistributional HR for change to HD (Table 5C).

### Technique Failure

If the analysis was performed for the outcome “technique failure,” including both death and change to HD, no significant association with BMI, LTI, and FTI could be observed (Supplementary Figure 3), also if this analysis was stratified by gender (Supplementary Figure 4).

Age, presence of cardiovascular diseases at baseline, overhydration, and use of polyglucose solutions were associated with an increased risk of technique failure compared with conventional solutions, and use of biocompatible solutions was associated with a lower risk of technique failure compared with conventional solutions, irrespective of whether the competing risk model was adjusted for BMI, LTI, or FTI (Supplementary Table 1).

## Discussion

This study showed associations of both patient- and prescription-related factors with body composition and its change over time. For the first time in a PD patient cohort, the use of BIS allowed a differentiated analysis of the association of PD prescription and evolution of lean and fat tissue mass, and of body composition and risk of death and change to HD. Although we found a *U*-shaped association between BMI and the risk of death, the body composition analysis allowed differentiation of high LTI being associated with a reduced risk of death, but high FTI being associated with an increased risk of death.

The participants in our study were recruited from different geographical regions, with varying treatment and prescription patterns (14). Accordingly, both APD and CAPD patients were represented. Furthermore, the options of the available PD solution portfolio were broadly utilized: type and strength of osmotic agent, solution buffer, and biocompatibility profile related to pH and presence of glucose degradation products (GDPs), although with some geographical disparity (14). Moreover, these prescription patterns were modified to some extent with time on PD, probably to adjust for a decrease in residual kidney function and change in peritoneal membrane function.

Assessment of nutritional status and body composition can be performed using various methods (19). In this study, we used BIS, a method also used in previous studies to evaluate body composition (20, 21). The distributions of LTI and FTI reported in our cohort coincide well with patterns found in other studies investigating prevalent HD (20), incident PD (22), or prevalent (23) PD populations, all of which measured body composition with the same method. It is obvious that body weight increases, primarily during the first year on PD, and to a minor extent further on. This is reflected in an increase of BMI, which is probably not associated with fluid overload because this decreases in the first year of PD (14). In the BrazPD cohort, which also included patients on incident PD, it was suggested that fluid overload rather than lean tissue or fat tissue was responsible for the weight gain in patients on incident PD. No bioimpedance data to assess body water, fat, and lean tissue were available in this cohort to confirm this, unfortunately (24).

In our study, as in other cohorts, fat tissue mass increased over time on PD (22), whereas lean tissue mass and thus LTI slightly decreased, resulting in a net gain in body weight not attributable to retention of water and sodium. Similarly, preservation of total protein despite increase in total body fat was found in previous studies (25).

Although it is impossible to disentangle nutritional status and inflammation, our cohort study reveals some interesting observations fitting the postulate that inflammation, malnutrition, and fluid overload are interlinked (26). LTI was higher and CRP concentration lower in categories with increasing serum albumin concentrations, and this at all time points. Both serum albumin and CRP are inflammatory markers, reacting in opposite directions during acute infection. In addition, serum albumin concentrations may correlate to albumin loss into the dialysate (27). Serum creatinine, the level of which increased over time, results from metabolization of creatine, a marker of muscle mass. Creatinine was indeed found to correlate with lean tissue mass (28) and with serum albumin (29). However, in our population, it increased over time more than lean tissue mass and albumin, indicating that it is both a marker of deterioration in renal creatinine excretion and of improved nutritional status.

Change of body composition over time on PD in our cohort was attributable to various factors, some of which are patient-associated and non-modifiable. Age, time on PD, and slow peritoneal membrane transport were significantly associated with a decrease in LTI over time, whereas age and time on PD were associated with an increase in FTI over time. In our cohort, PD prescription as a modifiable factor showed an association with changes in body composition over time, with a significant increase in FTI and a decrease in LTI over time associated with use of polyglucose or hypertonic solutions. This observed decrease of LTI is in line with an inverse correlation of prescribed glucose to change of LTI over time as described earlier (12). The association of an increase in LTI and decrease in FTI over the follow-up with use of biocompatible solutions was not statistically significant. In a small cross-sectional study, patients using biocompatible solutions with neutral pH and low GDP vs. conventional solutions had better nutritional markers and less systemic inflammation as reflected by lower CRP levels (30).

Both hypertonic glucose solutions and polyglucose solutions were used in an increasing proportion with time on PD, probably with the intention of enhancing ultrafiltration to compensate for decreasing residual diuresis. It is conceivable that a higher peritoneal absorption of carbohydrates resulted in an increase in body fat. However, there is conflicting data on the influence of glucose exposure on change in fat mass over time. One study observed, irrespective of glucose load, significant increases of dry body weight, BMI, adipose tissue mass, and FTI during the first year on PD, whereas lean tissue mass remained unchanged (22) in contrast to findings of another study (31). It remains inconclusive whether there is an association between glucose load and lipid profile changes, which was found in a study of patients with diabetes only (32), but not in studies of patients with and without diabetes (33, 34), and what long-term consequences might be. Data from the study by Pellicano et al. suggest that body fat may even be protective to limit protein wasting (25).

In an adjusted spline analysis, the subdistributional HR for technique failure is given over the whole range of observed values for body composition. For BMI, a *U*-shaped course of the HR of death could be observed, with increased mortality risk for a certain range of high and low BMI values. In contrast, no significant association with the risk of change to HD was observed, except for very high BMI values. A so-called reverse epidemiology, i.e., a decreased mortality risk associated with high BMI, has been found for the patients on HD (5, 35). This could not be confirmed by our data or in other studies on populations of patients on PD (8). Moreover, it was not clear from previous studies whether adiposity or increased muscle mass confers improved survival. In our study, with the use of BIS, we could for the first time differentiate the impact of lean tissue and fat tissue on patient outcomes and technique failure in PD and assess the ranges of these values and of BMI being predictive for an increased or decreased risk. This analysis confirms a reduced mortality risk for having an LTI higher than the median, whereas having a low LTI, thus a wasting state, is associated with an increased mortality risk. For FTI, this is complementary, with having values higher than median being associated with an increased mortality risk. From this, it could be derived that increased muscle mass rather than adiposity contributes to the improved survival in patients on PD with higher BMI. The association of nutritional markers with patient outcomes suggests that therapeutic plans should take into account the impact on body composition and apply dietary measures at an early stage (36, 37) with the aim of avoiding the loss of lean tissue mass and improving patient outcomes (38). With regard to change in treatment modality, the nutritional status seems not to be a trigger, as both LTI and FTI were not associated with the risk of change to HD. Here, other factors, such as loss of residual kidney function and limited peritoneal ultrafiltration, which might lead to overhydration, are more likely reasons for modality changes, as underlined by the observed association of the use of polyglucose or hypertonic PD fluid with change to HD.

Overhydration was borderline associated with the risk of death or risk of change to HD in the model, with adjustment for BMI, LTI, or FTI. In an analysis where both outcomes were combined, a significant association of overhydration with technique failure as defined by death or change to HD was observed, confirming our previous analysis (39) and the findings of other studies (40). Therefore, strategies for fluid status monitoring and early interventions might support the duration and clinical effectiveness of PD. Use of biocompatible (i.e., neutral pH and low GDP) solutions were associated with a lower risk of change to HD, but not with death. Longer maintenance on PD with biocompatible PD fluids might also be explained by better preservation of residual kidney function, but whether other parameters may also contribute, such as ultrafiltration, peritonitis occurrence, or hospitalization, remain an open question at the current stage of available evidence (41). The same meta-analysis also addressed death as an outcome and found no significant association with death for the use of biocompatible solutions (41).

Our study has several limitations. The study was designed as an observational study, and therefore, it is only possible to derive associations. It was beyond the scope of the study to also monitor dietary habits, thus their influence and that of changes over time could not be considered in our analyses.

In conclusion, body composition is associated with non-modifiable, patient-associated factors, and with modifiable treatment-related factors. The latter suggests adjusting prescriptions accordingly and monitoring body composition regularly to improve nutritional markers and, ultimately, control the risk of technique failure and improve patient outcomes.

## Data Availability Statement

The raw data supporting the conclusions of this article will be made available by the authors upon reasonable request, without undue reservation.

## Ethics Statement

The studies involving human participants were reviewed and approved by Ethics Committees and/or national authorities for the 135 study centers individually in accordance with national regulations. The patients/participants provided their written informed consent to participate in this study.

## Author Contributions

CV, CR, WV, TD, and AG designed the study. CV, CR, WV, JH, FV, AG, SA, and TD interpreted the results. AG, SA, and TD drafted the manuscript. CV, CR, WV, JH, FV, MV, IP, and RA acquired the data. All authors revised and approved the final version of the manuscript.

## Funding

This study was funded by Fresenius Medical Care Deutschland GmbH and Fresenius Medical Care Asia Pacific Ltd. The sponsor was involved in study design, data collection, analysis, and interpretation; preparation of the study report; and preparation of the manuscript.

## Conflict of Interest

TD, AG, and SA are full-time employees of Fresenius Medical Care. WV, FV, and JH received travel grants and speaker fees from Fresenius Medical Care and Baxter Healthcare. MV received grants from Fresenius Medical Care, Baxter, Amgen, and Vifor to attend conferences and scientific meetings. IP received travel grants from Fresenius Medical Care, Baxter Healthcare, Amgen, and Roche, as well as speakers' fees from Baxter Healthcare, Roche, and Amgen. The remaining authors declare that the research was conducted in the absence of any commercial or financial relationships that could be construed as a potential conflict of interest.

## Publisher's Note

All claims expressed in this article are solely those of the authors and do not necessarily represent those of their affiliated organizations, or those of the publisher, the editors and the reviewers. Any product that may be evaluated in this article, or claim that may be made by its manufacturer, is not guaranteed or endorsed by the publisher.

## Abbreviations

APD: Automated peritoneal dialysis
BCM: Body composition monitor
BIS: Bioimpedance spectroscopy
BMI: Body mass index
CAPD: Continuous ambulatory peritoneal dialysis
FTI: Fat tissue index
GDP: Glucose degradation product
HD: Hemodialysis
IPOD-PD: Initiative for patient outcomes in dialysis—peritoneal dialysis
IQR: Interquartile range
LTI: Lean tissue index
PD: Peritoneal dialysis.
