# Supplementary material for: Association of Prescription With Body Composition and Patient Outcomes in Incident Peritoneal Dialysis Patients
Source: Front Med (Lausanne). 2021 Dec 24;8:737165. doi: 10.3389/fmed.2021.737165 (PMC8738083; doi:10.3389/fmed.2021.737165)
Supplement: Supplementary file 1 [file Data_Sheet_1.docx]

**Verger et al. Association of prescription with body composition and patient outcomes in incident peritoneal dialysis patients**

**Supplemental Material**

**Supplemental Table 1:** Competing risk analysis on the influence of covariates together with BMI, LTI, FTI on the event ‘technique failure’ including both ‘death’ and ‘change to HD’; BL: Baseline

|  |  |  | **BMI** | | **LTI** | | **FTI** | |
| --- | --- | --- | --- | --- | --- | --- | --- | --- |
| **Factor** | **Category** | **Reference** | **Hazard**  **ratio** | **p-value** | **Hazard**  **ratio** | **p-value** | **Hazard**  **ratio** | **p-value** |
| Age (per 10 years) |  | Per 10 yrs | 1.020 | <0.001 | 1.018 | <0.001 | 1.018 | <0.001 |
| Gender | Female | Male | 0.999 | 0.994 | 0.881 | 0.382 | 0.938 | 0.628 |
| Diabetes (BL) | Yes | No | 1.024 | 0.854 | 1.024 | 0.855 | 1.000 | 0.998 |
| Cardiovascular (BL) | Yes | No | 1.351 | 0.023 | 1.360 | 0.020 | 1.323 | 0.034 |
| Liver disease (BL) | Yes | No | 1.483 | 0.132 | 1.412 | 0.186 | 1.480 | 0.134 |
| Overhydration (L) at Month 1 |  | Per 1 L | 1.100 | 0.001 | 1.090 | 0.004 | 1.111 | 0.001 |
| Modality (Month 1) | CAPD | APD | 0.906 | 0.495 | 0.869 | 0.332 | 0.903 | 0.480 |
| Hypertonic agent (Month 1) | Yes | No | 1.118 | 0.366 | 1.159 | 0.226 | 1.096 | 0.456 |
| Polyglucose use (Month 1) | Yes | No | 1.429 | 0.030 | 1.554 | 0.007 | 1.434 | 0.028 |
| Biocompatible solution (Month 1) | Yes | No | 0.580 | <0.001 | 0.583 | <0.001 | 0.590 | <0.001 |

**
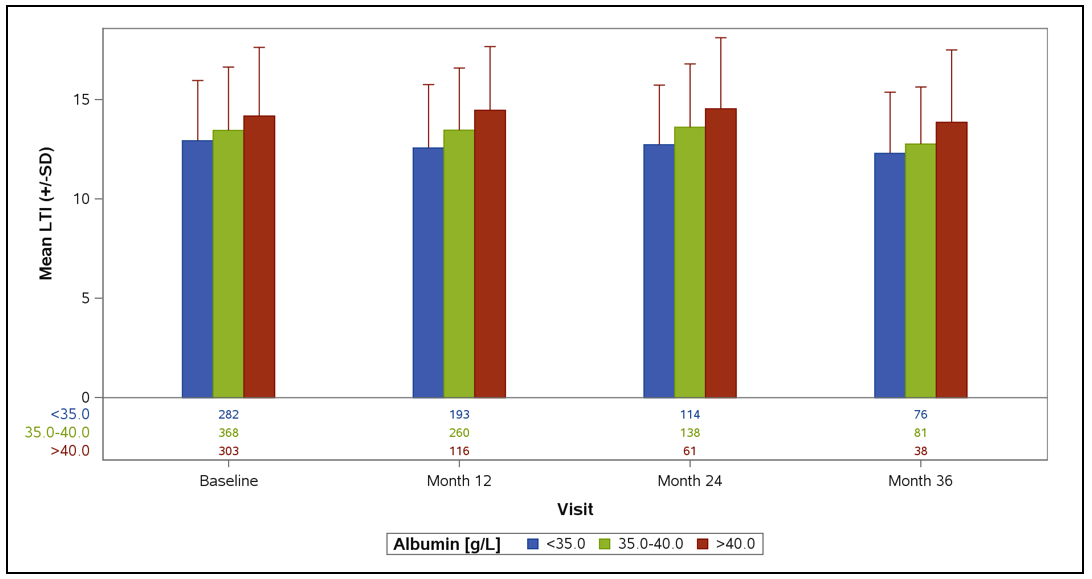
**

**Supplemental Figure 1:** Lean tissue index by categories of serum albumin and time on PD

**
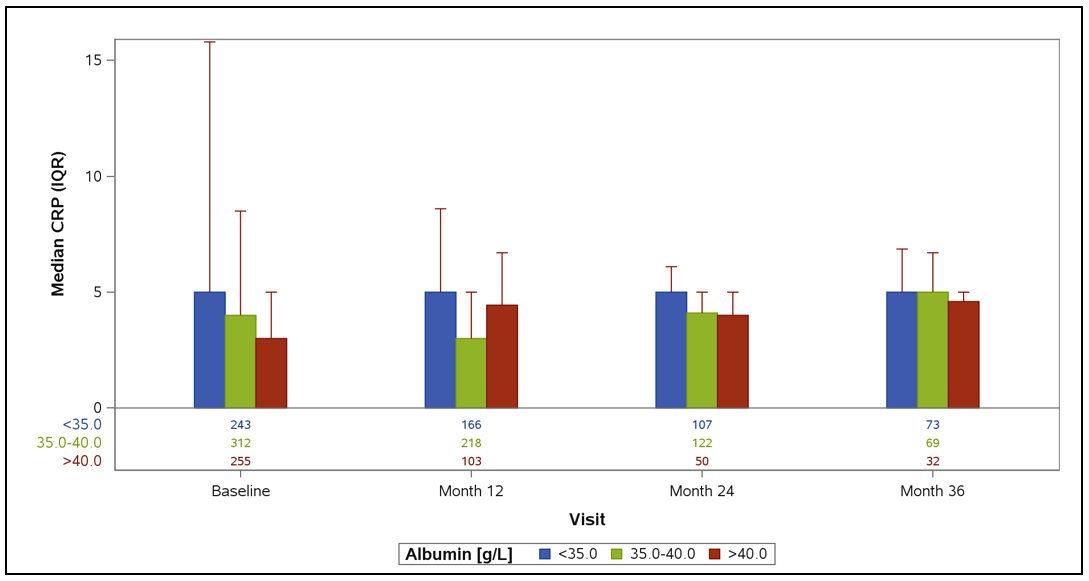
**

**Supplemental Figure 2:** CRP by categories of serum albumin and time on PD

**
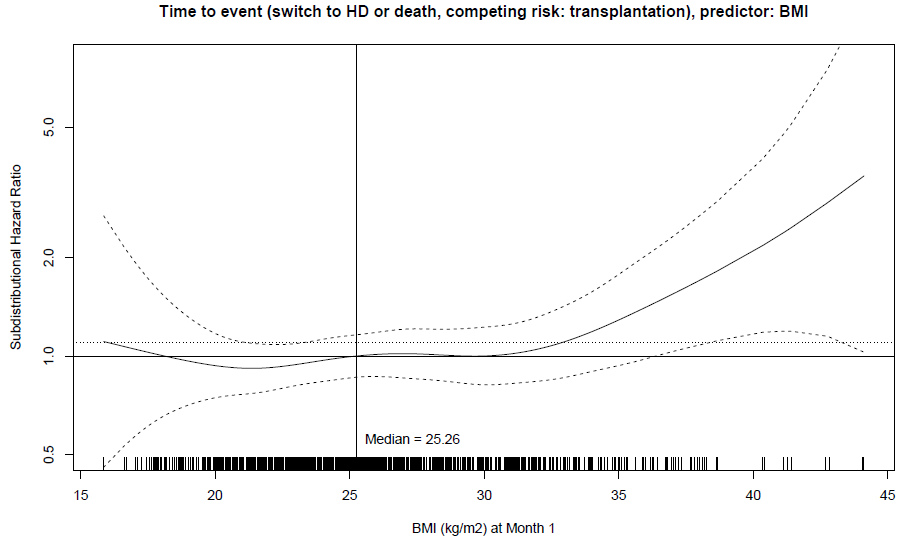
**A:

**
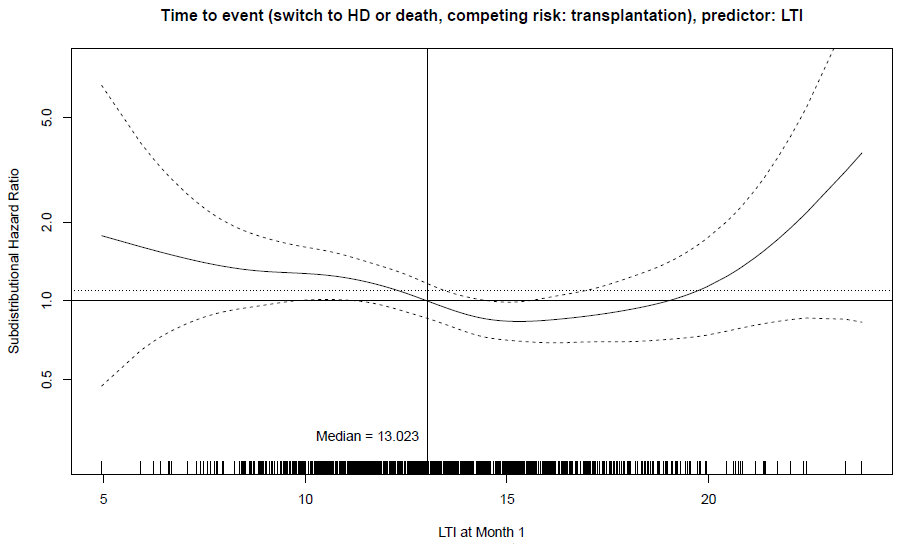
**B:

**
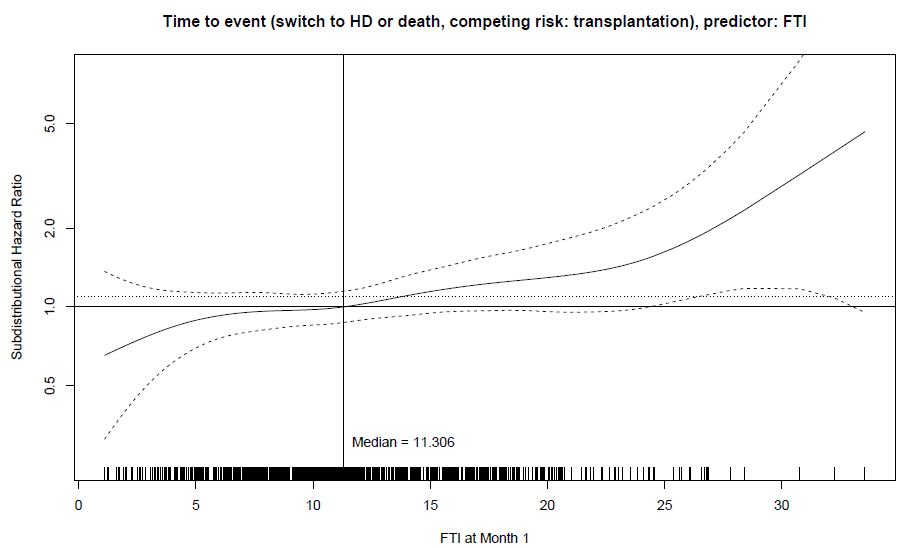
**C:

**Supplemental Figure 3**: Adjusted spline analysis for the association between BMI (A), LTI (B), FTI (C) and the event ‘technique failure’ including both ‘death’ and ‘change to HD’. Displayed is the subdistributional hazard ratio and confidence intervals across different BMI, LTI, FTI levels. Adjustment was performed for age, gender, comorbidities (diabetes mellitus, cardiovascular disease, liver disease), PD modality and PD solution types.

A:


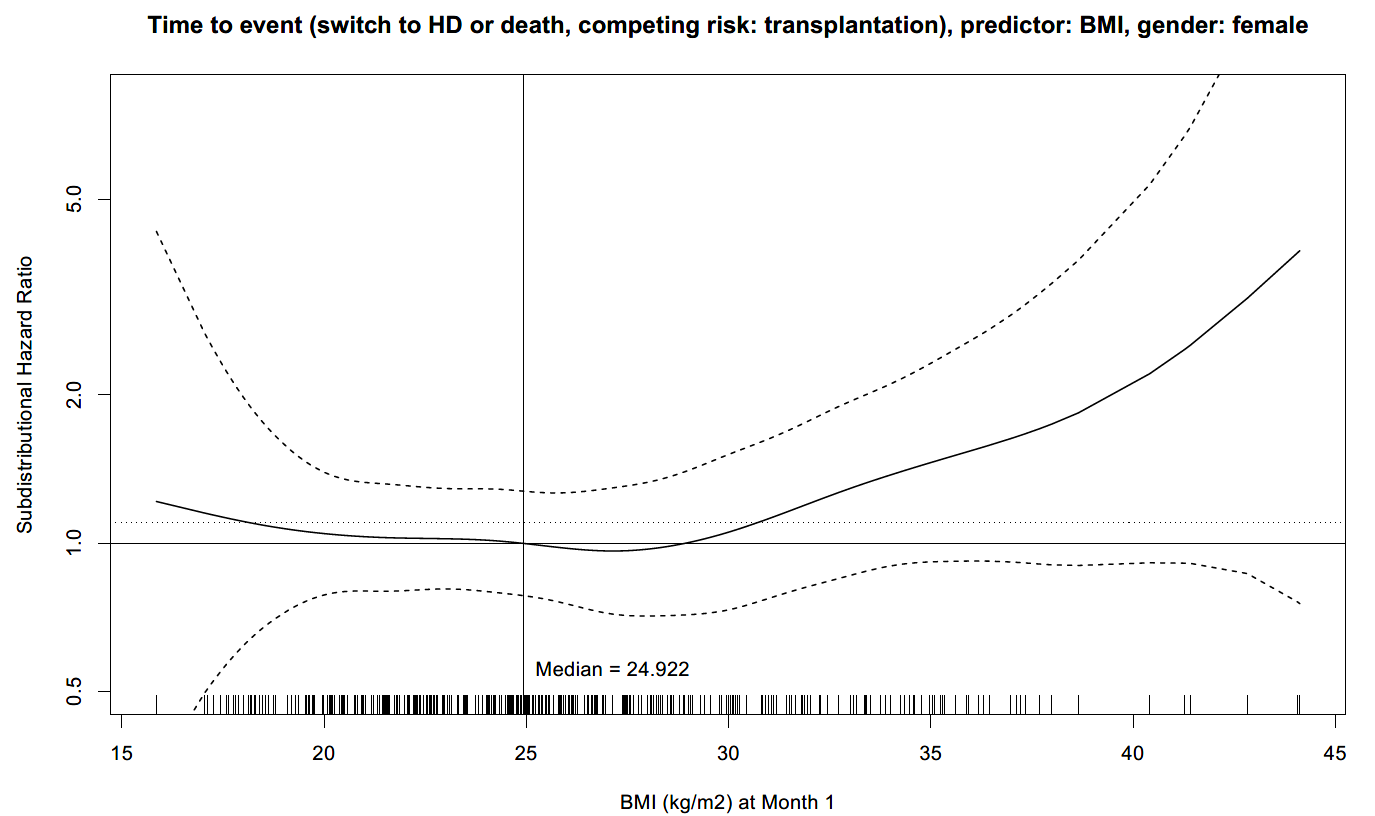

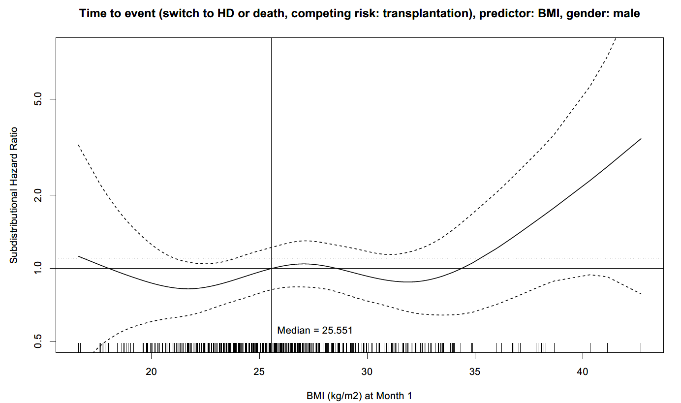


B:


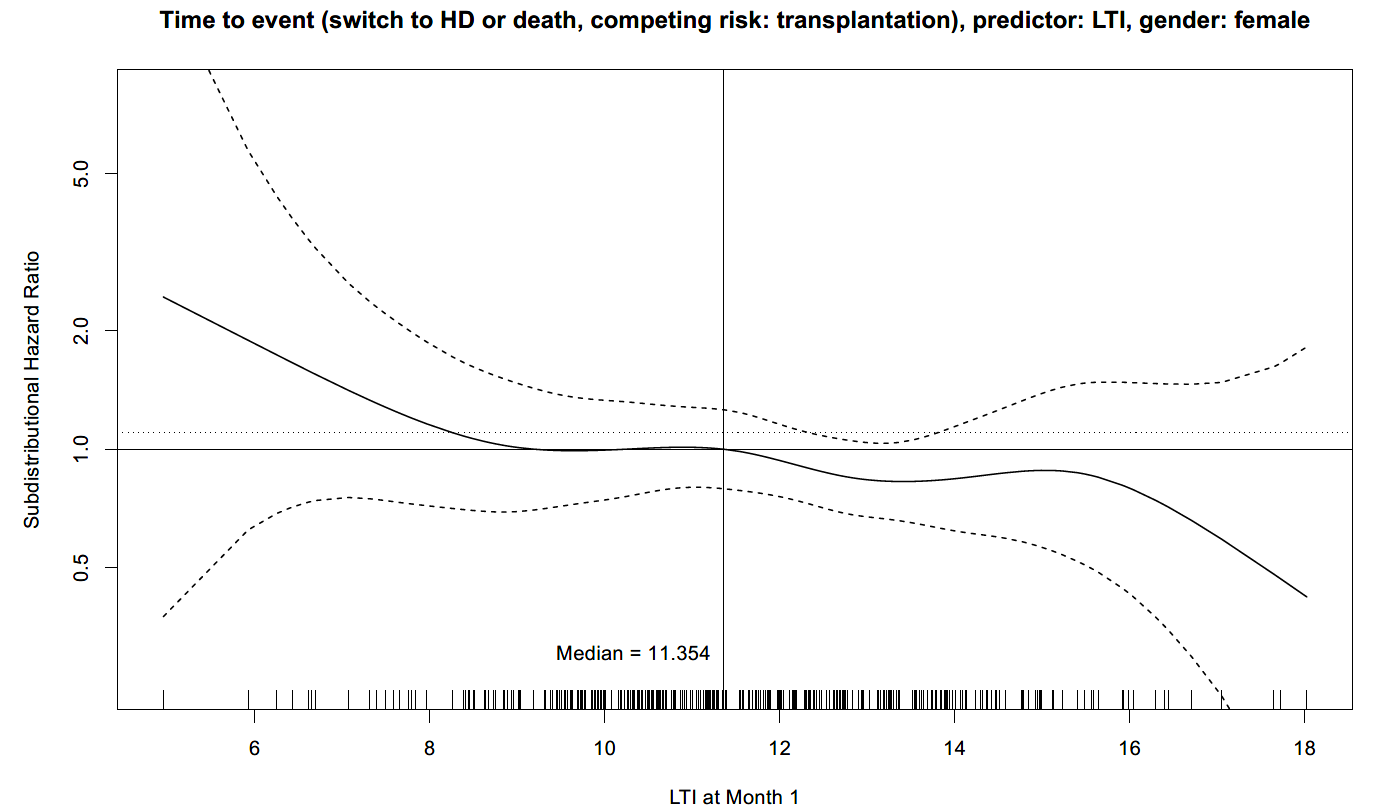

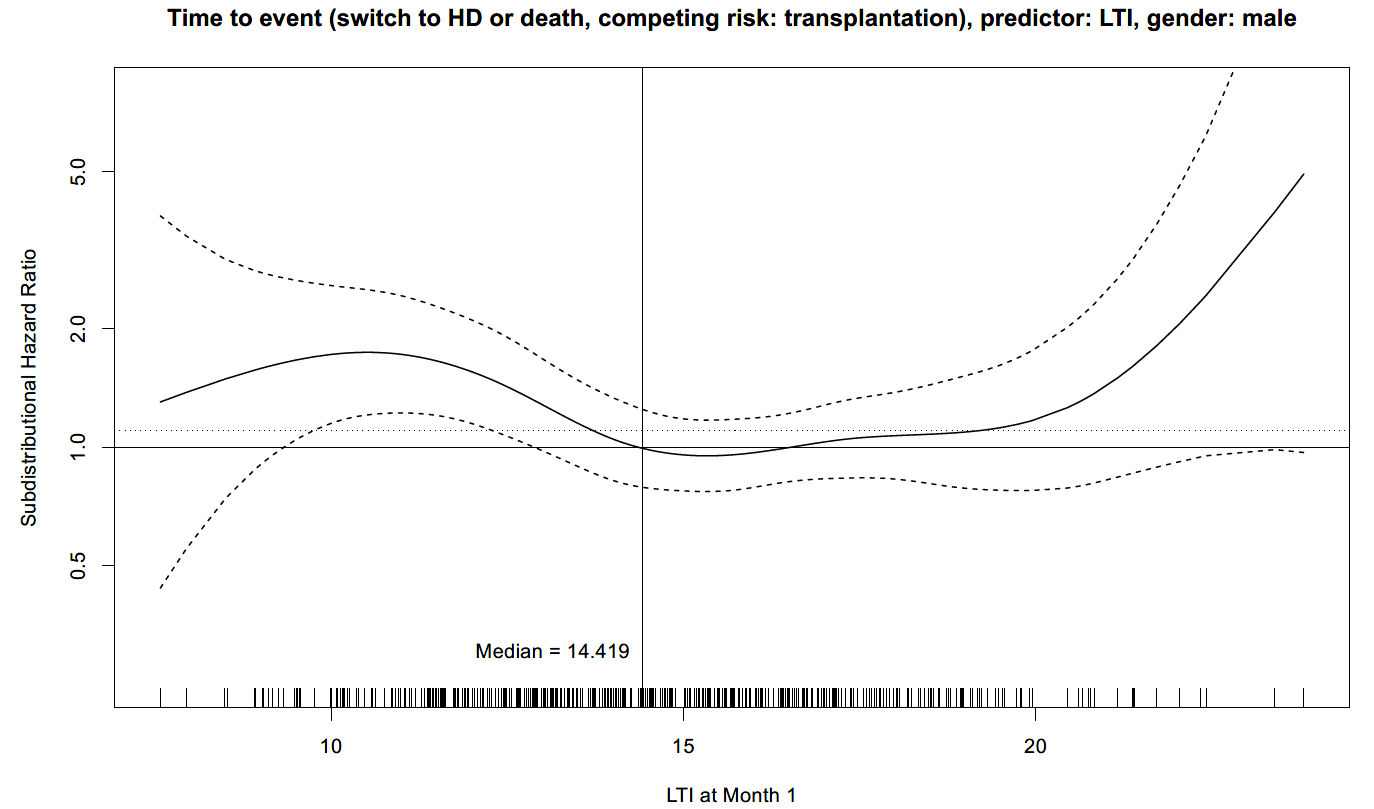


C:


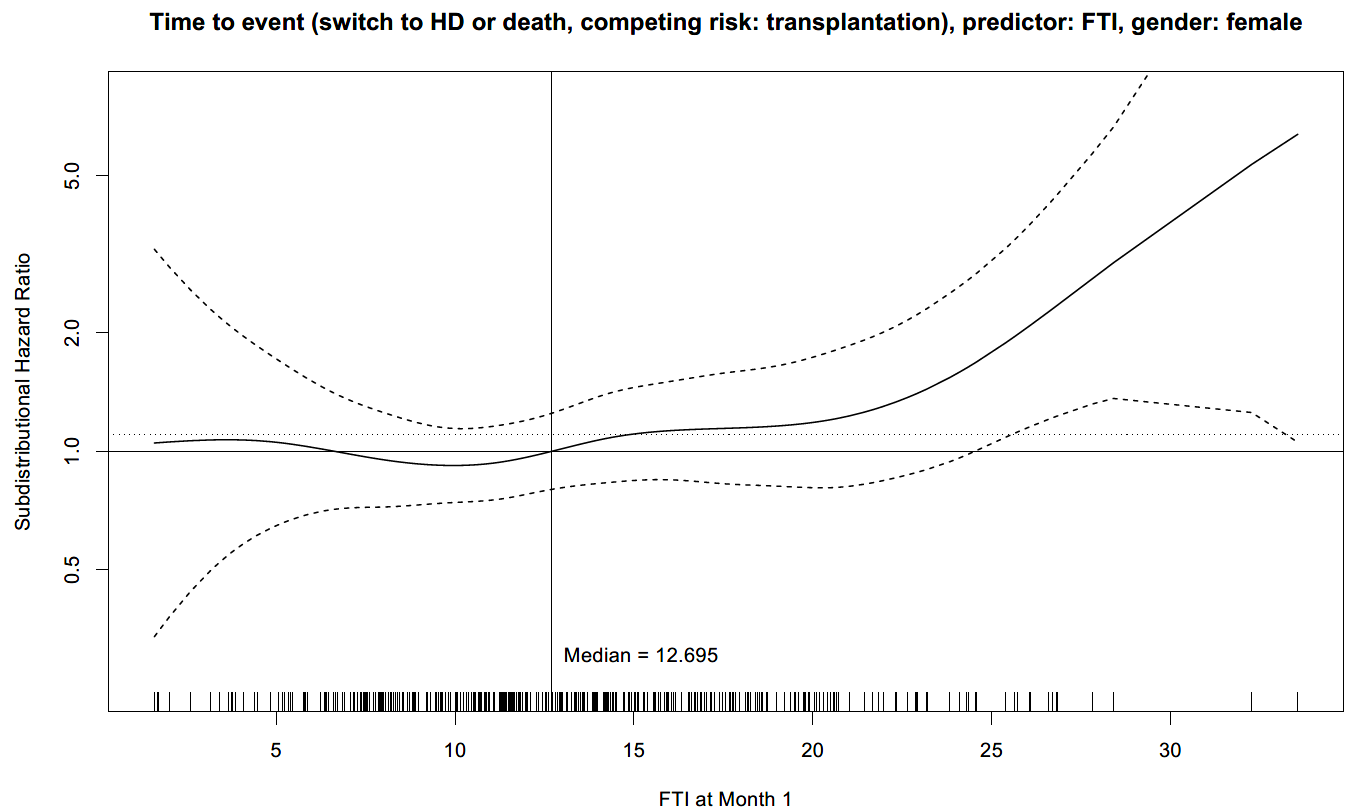

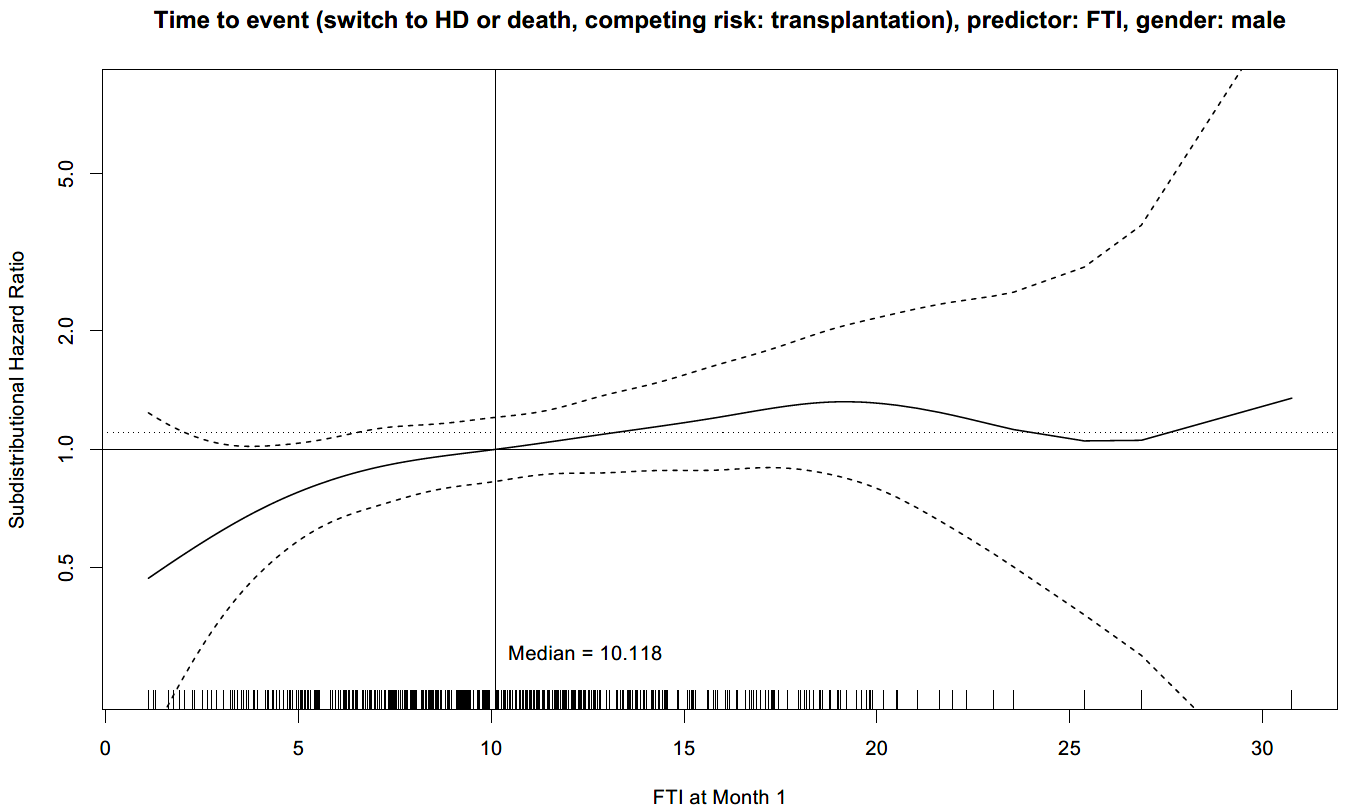


**Supplemental Figure 4:** Adjusted spline analysis for the association between BMI (A), LTI (B), FTI (C) and the event ‘technique failure’ including both ‘death’ and ‘change to HD’ by gender (left panels: male; right panels: female). Displayed is the subdistributional hazard ratio and confidence intervals across different BMI, LTI, FTI levels. Adjustment was performed for age, comorbidities (diabetes mellitus, cardiovascular disease), and PD solution types.
